# Evaluation of intraoperative microwave coagulo-necrotic therapy (MCN) for hepatocellular carcinoma: a single center experience of 719 consecutive cases

**DOI:** 10.1007/s00534-012-0527-5

**Published:** 2012-06-19

**Authors:** Yuko Takami, Tomoki Ryu, Yoshiyuki Wada, Hideki Saitsu

**Affiliations:** Department of Hepato-Biliary-Pancreatic Surgery, Clinical Research Institute, National Hospital Organization Kyushu Medical Center, 1-8-1, Jigyohama, Chuo-ku, Fukuoka, 810-8563 Japan

**Keywords:** Microwave coagulo-necrotic therapy, Microwave ablation, Hepatocellular carcinoma, Radiofrequency ablation, Hepatic resection

## Abstract

**Background:**

Hepatic resection (HRx) or radiofrequency ablation may be carried out as the first-line treatment of hepatocellular carcinoma (HCC). However, we have used intraoperative microwave ablation, named microwave coagulo-necrotic therapy (MCN) as part of our strategy for the treatment of HCCs for more than 15 years. Here we describe the treatment outcomes achieved at our institution as a high-volume center for microwave ablation.

**Methods:**

Between July 1994 and December 2010, 719 consecutive patients received MCN as their initial therapy for HCC (mean tumor size 26.9 mm, mean number of lesions 2.51) at our institute. The therapeutic survival effect, local tumor progression, and overall recurrence were prospectively evaluated and statistically analyzed.

**Results:**

The 1-, 3-, 5-, 7-, and 10-year overall survival rates of all 719 patients were 97.7, 79.8, 62.1, 45.3, and 34.1 %, respectively. Thirty percent of the patients had Child–Pugh class B cirrhosis and the 5-year survival rate of these patients was 46.6 %. The 5-year survival of patients with ≥4 lesions (*n* = 168) was 49.6 % and that of patients meeting the Milan criteria (*n* = 470) was 70.9 %. The 1-, 3-, 5-, 7-, and 10-year overall survival rates for 390 patients treated with MCN who had ≤3 lesions with diameter ≤3 cm were 97.9, 85.1, 70.0, 57.1, and 43.0 %. No significant differences were found between the overall survival rates after MCN and the overall survival rates in 34 patients treated with HRx during the same period at our institute and under the same (*P* = 0.3592), nor were there any differences in disease-free survival (*P* = 0.3496) and local recurrence rates between the MCN and HRx groups (*P* = 0.5926).

**Conclusion:**

MCN is effective for the locoregional control of HCC, with results comparable to those of HRx. MCN should be considered as one of the first-choice treatments for HCC, even for patients with poor liver function or multiple lesions.

## Introduction

Hepatic resection (HRx) has been considered as the most effective and radical treatment for hepatocellular carcinoma (HCC) [1]. However, HCC is usually associated with chronic hepatitis or liver cirrhosis, so most patients with HCC are not candidates for HRx owing to their poor liver function. For this reason, techniques that can eradicate the tumor and also preserve liver function are needed. One of these techniques, liver transplantation, has been used, but organ shortage limits its applicability.

More recently, some techniques for locoregional treatment have been devised. These minimally invasive ablation techniques, such as percutaneous ethanol injection therapy (PEIT) [2], cryosurgery [3], and radiofrequency ablation (RFA) [4], have been used to treat HCC. In particular, RFA has become the most widely used procedure in the past 10 years, mainly being employed by internists and radiologists. Long-term survivals after RFA have been reported recently [5, 6]. Additionally, it has been reported recently that the prognosis of patients with small HCCs treated with RFA is comparable to the prognosis in those treated with HRx [7].

Microwave ablation for HCC was developed before RFA in Japan [8–10], but for a while, it was replaced by the arrival of RFA. Although RFA is now used widely, we have opted for microwave coagulo-necrotic therapy (MCN) for locoregional therapy with surgical approaches since 1994, and the procedure for MCN is well established. Here, we report our 15 years of experience and the long-term results of MCN as first-line treatment, and evaluate the advantages of MCN.

## Patients and methods

### Patients

This survey was a cohort study conducted as a retrospective analysis of a prospective database at a single center. Between July 1994 and December 2010, 2297 patients underwent hepatic surgery at our institute. Of these, 938 patients underwent initial therapy for HCC. MCN was performed in 719 patients, HRx in 157, and HRx + MCN in 57. The remaining five patients were treated with laparotomic ethanol injection for tumors located near the hilum to prevent microwave thermal injury of the bile ducts.

### Selection of treatment method

For patients with HCC of ≤3-cm diameter, MCN is preferentially chosen for the treatment of fewer than ten tumors or thereabouts, regardless of liver function. However, if small numbers of tumors are located in regions suitable for liver resection, e.g., the surface or edge of the liver, HRx is sometimes performed.

Conversely, for patients with HCC of >3-cm diameter, HRx will be selected in the first instance. Nonetheless, even if the tumor is large, MCN will generally be selected for a patient with very poor liver function or an elderly patient with poor performance status.

For multiple HCCs consisting of more than ten nodules, surgical treatments such as HRx or MCN would not be selected, and other treatments, such as transcatheter arterial chemo-embolization (TACE) and hepatic arterial infusion (HAI) would be sought.

### Diagnosis of HCC

Histological confirmation of tumor tissue taken from biopsied samples after MCN was attempted for all patients. Six hundred and eleven patients were diagnosed to have HCC by histology. Heat degeneration after coagulation of the tumor tissue sometimes made it difficult to properly diagnose the disease in the remaining 108 patients; therefore, their HCC diagnoses were made on the basis of reliable clinical criteria fulfilling the following conditions: an appropriate clinical background (association with viral hepatitis, liver cirrhosis, or alcoholism); an increase in HCC-related tumor markers (namely, serum α-fetoprotein [AFP], lectin-reactive α-fetoprotein [AFP-L3], and plasma des-γ-carboxy-prothrombin [DCP] levels); and findings of high attenuation on the arterial phase and low attenuation on the portal phase (high-low pattern) of dynamic computed tomography (CT) or magnetic resonance imaging (MRI).

### MCN

Microwaves were generated by a Microtaze generator (Alfresa Pharma, Osaka, Japan) at a frequency of 2450 MHz. Microwave energy, conducted to an electrode, penetrated a few centimeters into the tissue and caused the tissue to generate heat by changing the polarity of the water molecules. This heat was not emitted externally but was generated in the tissue itself.

With a single coagulation procedure using a 16-gauge 150-mm-long needle (NESCO PERCUPRO-DP, MD-16CBP-1002150, Osaka, Japan) at 65 W for 1 min, a coagulo-necrotic area of about 1.0 cm in diameter and 1.5 cm long was formed around the end of the needle. With a single coagulation using a 21-gauge short needle at 80–85 W for 30 s, we formed a columnar necrotic area of about 1.0 cm in diameter and a length that was appropriate to the needle length. We used these two different types of needle to suit the particular conditions of the tumor being treated.

The necrotic area formed with single microwave irradiation was very small. Therefore, repeated electrode insertion and irradiation was required to obtain a sufficiently large treated area. Accordingly, MCN was not performed percutaneously, and we had three routes of approach to the tumor. If the tumor was located in the dome of the liver, a short thoracotomy was performed, and MCN was performed via the diaphragm. If the tumor was located on the surface of the liver, MCN was done under laparoscopy. Under other circumstances, MCN was performed at laparotomy.

After the approach to the tumor and before MCN was performed, the target lesion was defined under intraoperative sonography (Nemio 17; Toshiba, Tokyo, Japan) and the location and size of the tumor, as well as the number of nodules, were evaluated. We used a T-type probe (model PVF-738H, Toshiba, Tokyo, Japan) at open surgery, a convex-type probe (PVF-74 V, Toshiba, Tokyo, Japan) in a narrow space, and a probe for laparoscopy (PVM-787LA, Toshiba, Tokyo, Japan) at small thoracotomy. If the tumor could be seen on the surface of the liver, then the short-needle electrode, of a length that was appropriate for the tumor diameter, was inserted directly without ultrasound (US) guidance. If the tumor was located deeper in the liver, the long-needle electrode was inserted under US guidance. Under US guidance, needle insertion was better without using an adaptor than with one, because, using a free-hand method, the operator could control the angle or the depth of the electrode at his/her disposal. Electrodes needed to be inserted many times, in the vicinity of the tumor edge; moving toward the tumor center with each insertion. All ablations were carried out under real-time US monitoring. To secure a safe operative field, we apply a gauze pad between the region treated by MCN and other organs, and after ablation we cool the MCN region with cold saline.

Under US, as the coagulation was carried out, the air bubble produced by the local high temperature could be seen as a highly echoic dot. When the high-echoic area was widespread throughout the tumor and periphery with a margin of about 1 cm, we considered that complete ablation could be achieved. From various angles, we checked for the persistence of low-echoic areas and performed more coagulation if such areas were detected.

One week after the MCN, CT was performed to evaluate the ablation area and monitor for the presence of viable tumor remains, fluid collection, and abscess formation.

### Follow up

Follow-up US and measurements of serum AFP, AFP-L3, and DCP levels were performed every 2 months. Dynamic CT or dynamic MRI was performed every 3–5 months. The clinical observation periods following treatment ranged from 1 to 184 months (mean: 45.8 months).

### Treatment for recurrence

Local recurrence was defined as tumor progression adjacent to the treated site. Other recurrence in the liver was assessed as intrahepatic metastasis or multicentric carcinogenesis.

When recurrent tumors were found, regardless of the type of recurrence, if MCN or HRx could be attempted again, based on the same criterion as that used for the selection of treatment for the initial HCC, the procedures were repeated as many times as possible. However, HRx or MCN were thought to be unsuitable for multiple recurrences with roughly more than 10 nodules; thus, in such cases, TACE and HAI were applied. At present, liver transplantation is not a common treatment option in Japan, but transplantation from living donors was finally carried out in four patients. Additionally, since May 2009 we have used sorafenib as a molecular targeting therapy for multiple recurrences or distant metastases.

### Statistical analysis

Groups were compared using the unpaired *t*-test for continuous variables and Fisher’s exact test or the χ^2^ test for categorical variables. Overall survival was defined as the interval between the initial treatment and death or the date of the last or most recent follow-up visit. Disease-free survival was defined as the interval between the initial treatment and the date of the first treatment for recurrence. The survival curves were calculated by the Kaplan–Meier method and compared by a log-rank test. *P* < 0.05 was considered statistically significant. Univariate analysis was performed to identify clinical, biological, and tumor factors that predicted overall survival in patients with ≤3 tumors that were ≤3 cm in size. A Cox proportional hazards model was fitted to each of the variables. All variables with *P* < 0.05 by univariate comparison were subjected to multivariate analysis to assess their value as independent predictors. Results of univariate and multivariate analyses were presented as hazard ratios with corresponding 95 % confidence intervals (CIs), with the *P* value. Data analysis was performed with Stat View J 5.0 computer software (SAS Institute, Cary, NC, USA).

## Results

We analyzed 719 consecutive patients with HCC (mean tumor size: 26.9 ± 11.9 mm, mean tumor number: 2.51 ± 2.04) treated by MCN alone. The baseline characteristics of the patients are presented in Table 1. Hepatitis C accounted for about 73.0 % of all the background liver diseases, and 216 patients had poor liver function and Child–Pugh class B cirrhosis. In all, 335 patients had one nodule, 216 had two or three, and 168 had ≥4 tumors. Five hundred and two patients had a tumor <3 cm in diameter and 217 had a tumor ≥3 cm in diameter.

**Table 1:** Characteristics of 719 patients treated with MCN

Characteristic	No. or mean ± SD
Sex (male:female)	474:245
Age (years)	67.6 ± 9.1
Hepatitis (B/C/BC/no B no C)	92/525/11/91
Serum albumin (g/dl)	3.7 ± 0.5
Total bilirubin (mg/dl)	1.0 ± 0.7
Prothrombin activity (%)	79 ± 15
Child–Pugh class (A/B/C)	503/216/0
Tumor size (mm)	26.9 ± 11.9
Range(mm)	7.9–80.0
Tumor <2 cm, *n* (%)	201 (28.0)
2 ≤ Tumor < 3 cm, *n* (%)	301 (41.9)
3 ≤ Tumor < 5 cm, *n* (%)	182 (25.3)
Tumor ≥5 cm, *n* (%)	35 (4.8)
Tumor number	2.51 ± 2.04
Range	1–12
1, *n* (%)	335 (46.6)
2 or 3, *n* (%)	216 (30.0)
≥4, *n* (%)	168 (23.4)
TNM stage by LCSGJ (I/II/III/IV)	111/317/279/12
Serum AFP (ng/ml)	614.9 ± 4411.5
L3 (%)	11.2 ± 18.5
DCP (mAu/ml)	675.6 ± 3376.0

Data are given as means ± standard deviation

*LCSGJ* Liver Cancer Study Group of Japan, *AFP* serum α-fetoprotein, *L3* lectin-reactive α-fetoprotein, *DCP* plasma des-γ-carboxy-prothrombin

### All patients treated with MCN

**Fig. 1:**
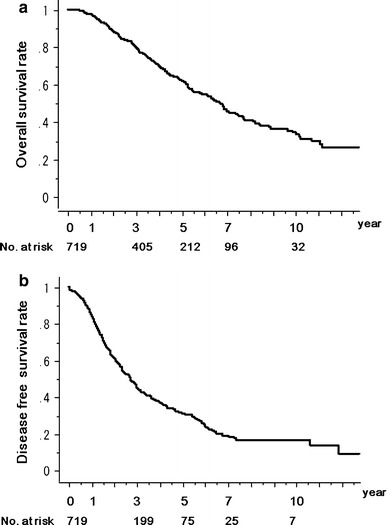
**a** Kaplan–Meier overall survival rate and **b** Kaplan–Meier disease-free survival rate for 719 patients who received microwave coagulo-necrotic therapy (MCN). The number of patients at risk at each time point is shown *below the graphs*

The overall survival rates for all patients treated with MCN were 97.7, 79.8, 62.1, 45.3, and 34.1 %, at 1, 3, 5, 7, and 10 years, respectively (Fig. 1a). The 1-, 3-, 5-, 7-, and 10-year disease-free survival rates were 84.6, 45.0, 31.0, 18.8, and 16.5 %, respectively (Fig. 1b).

Computed tomography (CT) at 1 week after MCN showed that all nodules had been completely ablated at the time of the operation. None of the patients needed additional ablation immediately after the first treatment.

### Comparison of MCN with HRx

We compared the results of MCN and HRx in patients with ≤3 lesions of ≤3 cm diameter. Table 2 shows the patient and tumor characteristics of the two groups. There were no significant differences in patient characteristics.

**Table 2:** Characteristics of patients with ≤3 tumors ≤3 cm in diameter

Characteristic	MCN (*n* = 390)	HRx (*n* = 34)	*P* value
Sex (male:female)	236:154	26:8	0.0663
Age (years)	67.6 ± 9.1	66.1 ± 7.5	0.3478
Hepatitis (B/C/BC/no B no C)	49/289/6/46	4/20/3/7	0.0130
Serum albumin (g/dl)	3.72 ± 0.03	3.87 ± 0.55	0.1008
Total bilirubin (mg/dl)	1.03 ± 0.79	0.90 ± 0.09	0.3527
Prothrombin activity (%)	78.6 ± 15.6	82.6 ± 17.6	0.1553
Child–Pugh class (A/B/C)	273/117/0	27/7/0	0.2324
Tumor size (mm)	20.8 ± 5.0	22.3 ± 4.7	0.0878
Number of tumors	1.55 ± 0.75	1.11 ± 0.47	0.0012
TNM stage by LCSGJ (I/II/III/IV)	111/189/89/1	8/19/3/4	<0.0001
Serum AFP (ng/ml)	461.0 ± 5279.5	151.3 ± 312.6	0.7329
L3 (%)	10.0 ± 17.0	11.1 ± 21.4	0.7505
DCP (mAu/ml)	376.4 ± 2372.6	788.6 ± 3864.1	0.4060

Data are given as means ± standard deviation

*MCN* microwave coagulo-necrotic therapy, *HRx* hepatic resection, *LCSGJ* Liver Cancer Study Group of Japan, *AFP* serum α-fetoprotein, *L3* lectin-reactive α-fetoprotein, *DCP* plasma des-γ-carboxy-prothrombin

**Fig. 2:**
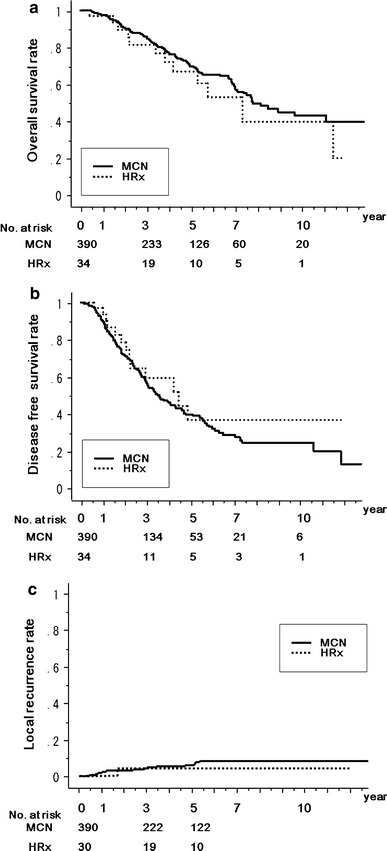
Comparison between MCN group (*n* = 390, *solid line*) and hepatic resection (*HRx*) group (*n* = 34, *dotted line*) in patients with ≤3 lesions ≤3 cm in diameter. **a** Kaplan–Meier estimation of overall survival, *P* = 0.3592. **b** Kaplan–Meier estimation of disease-free survival, *P* = 0.3496. **c** Kaplan–Meier estimation of local recurrence rate, *P* = 0.5926. The number of patients at risk at each time point is shown *below the graphs*

In patients with ≤3 lesions of ≤3 cm diameter, the overall survival rates for the 390 patients who were treated with MCN were 97.9, 85.1, 70.0, 57.1, and 43.0 % at 1, 3, 5, 7, and 10 years, respectively; and the rates in those treated with HRx were 97.1, 81.4, 66.9, 52.7, and 39.5 % (Fig. 2a). Disease-free survival rates at 1, 3, 5, 7, and 10 years, respectively, in the MCN group were 89.8, 56.3, 39.6, 28.4, and 24.4 %, and those in the HRx group were 97.0, 59.0, 36.9, 36.9, and 36.9 % (Fig. 2b). Local recurrence rates at 1, 3, and 5 years in the MCN group were 1.9, 4.8, and 5.9 %, respectively; and those in the HRx group were 0, 4.3, and 4.3 % (Fig. 2c). The overall survival (*P* = 0.3592) (Fig. 2a), disease-free survival (*P* = 0.3496) (Fig. 2b), and local recurrence (*P* = 0.5926) (Fig. 2c) rates were comparable after MCN and HRx. Under these tumor conditions, the choice of the treatment procedure of either MCN or HRx was not significantly associated with overall survival (*P* = 03608, univariate analysis). After multivariate analysis, serum albumin (*P* = 0.0498), Child–Pugh grade (*P* < 0.0001), tumor size (*P* = 0.0034), and number of tumors (*P* = 0.0491) were found to be independent predictors of overall survival (Table 3).

**Table 3:** Predictors of overall survival in patients with ≤3 tumors ≤3 cm in diameter

Characteristic	Univariate			Multivariate		
	Hazard ratio	95 % CI	*P* value	Hazard ratio	95 %CI	*P* value
Sex (male:female)	0.937	0.651–1.348	0.7270			
Age (years)	1.011	0.990–1.032	0.3075			
Hepatitis (B or C/no B no C)	1.922	0.938–3.937	0.0742			
HRx/MCN	1.320	0.727–2.376	0.3608			
Serum albumin	0.479	0.343–0.663	<0.0001	0.600	0.360–1.000	0.0498
Total bilirubin	1.102	0.930–1.307	0.2617			
Prothrombin activity	0.982	0.969–0.995	0.0058	0.991	0.976–1.006	0.6638
Child–Pugh class (A/B)	0.485	0.339–0.693	<0.0001	0.482	0.336–0.692	<0.0001
Tumor size (mm)	1.052	1.014–1.091	0.0069	1.057	1.019–1.098	0.0034
Number of tumors	1.345	1.077–1.679	0.0090	1.253	1.011–1.568	0.0491

Hazard ratios for overall survival were calculated by Cox proportional hazard regression analysis

*CI* confidence interval

### Results of patients treated with MCN under various tumor and host conditions

**Fig. 3:**
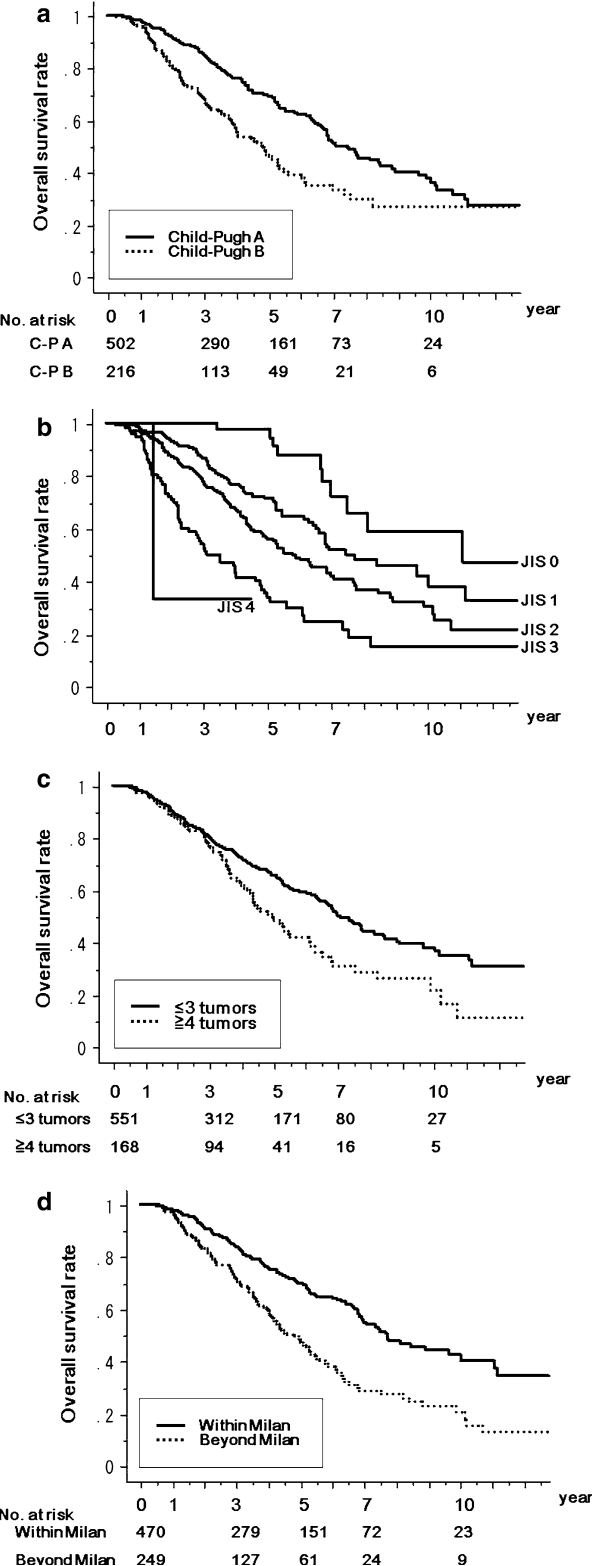
Kaplan–Meier survival estimations of patients treated with MCN under various conditions. The number of patients at risk at each time point is shown *below the graphs*. **a** Overall survivals among Child–Pugh class A patients (*C-P A*; *n* = 502, *solid line*) and class B patients (*C-P B*; *n* = 216, *dotted line*), *P* < 0.0001. **b** Overall survival according to the Japan Integrated Staging (*JIS*) score, *P* < 0.0001. **c** Overall survivals of patients with ≤3 tumors (*n* = 551, *solid line*) and those with ≥4 tumors (*n* = 168, *dotted line*), *P* = 0.0016. **d** Overall survival of patients who met the Milan criteria (*Within Milan*; *n* = 470, *solid line*) versus those who did not (*Beyond Milan*; *n* = 249, *dotted line*), *P* < 0.0001

First we assessed the overall survival of patients according to their liver function, using the Child–Pugh classification. Among the 719 patients, 30.2 % had Child–Pugh class B cirrhosis. The 1-, 3-, 5-, 7-, and 10-year survival rates of patients with class A (*n* = 502) were 98.3, 85.0, 69.2, 50.4, and 36.7 %, respectively, and in those with class B (*n* = 216), the rates were 96.1, 67.8, 46.6, 33.1, and 27.0 % (*P* < 0.0001) (Fig. 3a).

Overall survival was also assessed according to the Japan Integrated Staging (JIS) score. The 1-, 3-, 5-, and 10-year survival rates of patients with a JIS score of 0 (*n* = 74) were 100, 100, 97.6, and 58.5 %, respectively. The rates in those with a JIS score of 1 (*n* = 270) were 98.8, 86.8, 71.4, and 41.9 %, while the rates in those with a JIS score of 2 (*n* = 268) were 96.9, 78.6, 56.5, and 30.0 %, respectively. The rates in those with a JIS score of 3 (*n* = 104) were 94.9, 53.8, 35.2, and 15.0 %, respectively. The remaining three patients had JIS scores of 4, and the 3-year survival rate in these patients was 33.3 % (*P* < 0.0001) (Fig. 3b).

When we assessed the overall survival of patients according to the number of tumors treated with MCN, the 1-, 3-, 5-, 7-, and 10-year overall survival rates of patients with ≤3 lesions (*n* = 551) were 97.9, 80.4, 65.9, 49.8, and 37.9 %, respectively. The overall survival rates for patients with ≥4 lesions (*n* = 168) treated with MCN were 96.9, 77.7, 49.6, 30.7, and 21.7 % at 1, 3, 5, 7, and 10 year, respectively (*P* = 0.0016) (Fig. 3c).

The 1-, 3-, 5-, 7-, and 10-year survival rates of patients treated with MCN who met the Milan criteria (single HCC ≤5 cm or up to 3 nodules ≤3 cm) (*n* = 470) were 98.0, 84.6, 70.9, 57.2, and 45.6 %, respectively. The respective survival rates in MCN patients who did not meet the Milan criteria (*n* = 249) were 97.0, 72.3, 48.5, 28.6, and 20.2 % (*P* < 0.0001) (Fig. 3d).

Additionally, we assessed the survival and recurrence rates for each decade of the study period; no differences were observed between the 1990s and 2000.

### Complications of MCN

Major and minor complications of MCN occurred in 51 patients (7.0 %) (Table 4). Liver abscess occurred in four patients (0.6 %). Pleural effusion or ascites that required prolongation of hospital stay or drainage occurred in 13 patients (1.8 %). Delayed wound healing with surgical site infection was seen in 17 patients (2.4 %).

**Table 4:** Complications in 719 patients treated with MCN

Complication	No.
All	51
Delayed wound healing	17
Pleural effusion or ascites	13
Pneumonia or atelectasis	4
MCN abscess	4
Intraabdominal or intrathoracic bleeding	4
Cerebro-or cardiovascular events	3
Upper gastrointestinal bleeding	1
Others^a^	5

^a^Transcervical fracture, pulmonary infarction, fulminant hepatitis, choledocholithiasis, disseminated intravascular coagulation (DIC)

Fulminant hepatitis occurred in a 48-year-old male patient, who was positive for hepatitis B envelope (e) antigen and who did not receive treatment with the then-new anti-HBV therapy, lamivudine, in 2002. Although his HCC was a 15.2-mm small nodule located in the dome of the liver, and MCN itself was performed safely without unexpected accidents, some factor other than MCN (e.g., anesthesia or antibiotics) may have triggered the severe hepatitis.

## Discussion

MCN for the locoregional ablation of HCC has been used in Japan since 1988. In 1977, Tabuse [8] developed the microwave coagulator, Microtaze, to achieve hemostasis during hepatic resection. In 1988, using this device, Saitsu et al. [9] systematically started to coagulate not only the hepatic parenchyma, but also the tumor itself, and first reported the efficacy of this new treatment, named MCN, for the intraoperative and laparoscopic ablation of small HCCs in 1991. In 1999, Seki et al. [10] reported that percutaneous microwave coagulation therapy might be superior to PEIT for the local control of moderately or poorly differentiated small HCCs.

However, since 1995, when RFA was introduced, it has been used as an emerging technology for the treatment of HCC [11]. In a study by Shibata et al. [12] in 2002, the number of treatment sessions per nodule was reported to be lower in their RFA group than in their percutaneous microwave coagulation group. 12With fewer sessions required for needle insertion and ablation into the center of the nodule, RFA seems to result easily in a large homogeneous ablation volume. This probably explains why RFA has gained popularity worldwide.

However, in recent years, the potential advantages of MCN have been reviewed [13], and an increasing number of reports have shown that microwave radiation has some advantages over other modes of treatment for HCC [14–16]. Compared with RFA, microwave ablation has several theoretical advantages. First, microwave ablation has a much broader zone of active heating, whereas RFA heating is primarily passive, and does not rely on the conduction of electricity into tissue. Therefore, intratumoral temperatures with microwave ablation can be consistently high, which potentially contributes to complete tumor killing [17]. Second, because the cooling effect of blood flow is greatest within the zone of conductive rather than active heating, microwave ablation is less affected by the perfusion-mediated ‘heat-sink’ effect, and this may allow for more uniform tumor killing both within a targeted zone and next to blood vessels [18].

Additionally, we think that the greatest difference between MCN and other locoregional therapies is how to approach the tumor. All methods of percutaneous ablation therapy tend to start with puncture and ablation at the tumor center. However, MCN starts its irradiation from the tumor edge and moves towards the tumor center.

In 1999, we used RFA to treat HCCs in 66 patients. About 10 % of the patients experienced curious recurrences of HCC that expanded from the area of ablation to the peripheral segmental area of the liver. These strange intrahepatic recurrences after RFA were distinguished from the local recurrences that occur after RFA and needle-track tumor seeding, and were reported as multiple scattered recurrences or intrahepatic (metastatic) dissemination [19, 20]. Ruzzenente et al. [21] have reported this type of recurrence in 4.5 % of patients treated with RFA.

It has been speculated that an increase in intratumoral pressure, which results from the elevated temperature during the ablation process, may induce the release of tumor cells. Kotoh et al. [22] have shown an increase in pressure near the ablated area during RFA. Of note, Kawamoto et al. [23] monitored intrahepatic pressure in pigs with a LeVeen or cooled-tip needle and they concluded that increased intrahepatic pressure could be controlled using a multi-step method rather than a single-step method with the LeVeen or cooled-tip needle.

Electrode insertion and ablation at the center of a tumor causes an increase in intratumoral pressure. Consequently, tumor microthrombi that arise in the vessels adjacent to the tumor are extruded through the portal vein and appear in the peripheral liver parenchyma. Okusaka et al. [24] have shown microvascular invasion in 17 % of patients with a tumor <2 cm and in 20 % of patients with a tumor 2–3 cm in diameter. Even small HCC tumors can have microvascular invasion, which can be pushed out to the periphery of the tumor by the increase in intratumoral pressure. At the outset of the use of MCN in 1988, we made the first electrode insertion at the tumor edge and not in the center, and we performed insertions and coagulations 5–8 times, even for the treatment of a tumor <2 cm in diameter. This procedure could probably make it possible to avoid any unwanted elevation in intratumoral pressure, which we believe is the most important way to prevent tumor dissemination. We have not yet monitored intratumoral pressure during MCN, but we have not seen any of the curious recurrences that are seen with RFA.

Some recent studies have reported improvements in microwave antennae that lead to the achievement of larger coagulation zones [25, 26]. However, in terms of intratumoral pressure, we think that the tumor should not be ablated with one puncture and ablation into the center of the tumor, even with microwave ablation. In consideration of the foregoing oncological aspects, we have carried out MCN with a multiple insertion and coagulation method.

We initially compared the results of MCN and HRx prospectively in patients with ≤3 tumors that were ≤3 cm in diameter. The patient and tumor characteristics of the two groups did not differ significantly. We found that in this cohort, the overall survival, disease-free survival, and local recurrence rates after MCN were equivalent to those after HRx; therefore, we think that the locoregional control achieved with MCN is equivalent to that achieved with HRx. Also, the local recurrence rate of 1.9 % at 1 year after MCN was lower than the 2–15 % that was observed after RFA in tumors of the same size [27, 28].

Because of the surgical approach of MCN, MCN is thought to be more invasive than other percutaneous ablation methods. However, with the small volume of irradiation, it is possible to adjust the total ablated area and avoid ablation of the surrounding important structures, such as arteries and the biliary tract. Therefore, we think that MCN can preserve liver function after treatment, which is necessary in patients who have poor liver function. We analyzed the overall survival of patients stratified according to liver function, using the Child–Pugh class. The overall survival of patients with Child–Pugh class A was significantly better than the survival in those with class B, but we did achieve a 5-year survival of 46.6 % in class B patients. Patients with poor liver function are not usually indicated for surgical treatment, and are often treated with non-surgical methods such as TACE or HAI. However, with MCN, we can provide complete locoregional therapy with results comparable to those of HRx, even in patients with Child–Pugh class B disease.

Moreover, in the present study, the average hospital stay after MCN (13.5 ± 7.27 days) was significantly shorter than that after HRx (24.5 ± 24.97 days) (*P* = 0.0014). If any complications, including bile leak, occur following HRx in a Child–Pugh class B patient with a poor liver function score, healing tends to be very difficult and the hospital stay tends to be longer. Therefore, we recommend the use of the low invasive technique, MCN, particularly for the treatment of patients with poor liver function.

In addition to the Child–Pugh classification results, we assessed the results according to the JIS score. This system was proposed by Kudo et al. [29] and consists of the Child–Pugh classification and the TNM staging system of the Liver Cancer Study Group of Japan (LCSGJ); the JIS score is considered to be a good prognostic staging system for HCC and an important tool for comparisons between different therapeutic strategies. We were able to easily compare MCN with other modalities, with the results shown in Fig. 3b.

Another advantage of MCN is the ability to treat multiple nodules at the same time. Patients with multiple-tumor HCC are rarely indicated for surgical treatment, and generally these patients are treated with TACE or HAI. However, the 5-year survival rate for TACE and HAI is only about 16 % [30]. Under laparotomy, thoracotomy, or laparoscopy, we can ablate all the nodules seen in the operative field. Unfortunately, we have no data for the results of patients treated with TACE or HAI at our institution. However, the 5-year survival of patients with ≥4 lesions in our present study was 49.6 %; this result is better than those reported for TACE or HAI in previous studies [30].

In the present study, the 5-year overall survival of patients treated with MCN who met the Milan criteria was 70.9 %, without liver transplantation. Over a 5-year period, repeat MCN or HRx for recurrence was performed a mean of 1.72 times (range 1–8 times). Repeat treatment was needed, but in Japan, because there is a shortage of liver donors, this good outcome has proved the usefulness of MCN.

Finally, we assessed the complications of MCN. Intractable ascites or pleural effusion and delayed wound healing occurred frequently, probably because many patients had poor liver function. Others have reported perforation of other neighboring organs as a severe complication of MCN [31]. However, we have not yet experienced such a complication.

In conclusion, our results show that MCN can achieve good locoregional control of HCC, comparable to that achieved with HRx, without adverse effects on liver function. However, because MCN requires skill and expertise (for example, the ability to perform laparotomy and puncture under US guidance without a guideline adaptor), it has not yet been adopted worldwide. Nevertheless, we believe that MCN could be used as one of the first-choice treatments for HCC. In particular, MCN is well-suited for treating patients with reduced liver function and those initially presenting with multiple lesions.

## Abbreviations

HCC: Hepatocellular carcinoma
MCN: Microwave coagulo-necrotic therapy
RFA: Radiofrequency ablation

## References

[1] Takayama T, Makuuchi M, Hirohashi S, Sakamoto M (1998). Early hepatocellular carcinoma as an entity with a high cure rate of surgical cure. Hepatology.

[2] Majima Y, Tanikawa K (1991). Percutaneous ethanol injection therapy (PEIT) for small hepatocellular carcinoma smaller than 30 mm in diameter. Jpn J Med Ultrasonics.

[3] Pearson AS, Izzo F, Fleming RYD, Ellis LM, Delrio P, Roh MS (1999). Intraoperative radiofrequency ablation or cryoablation for hepatic malignancies. Am J Surg.

[4] Livraghi T, Goldberg SN, Lazzaroni S, Meloni F, Solbiati L, Gazalle GF (1999). Small hepatocellular carcinoma: treatment with radio-frequency ablation versus ethanol injection. Radiology.

[5] Tateishi R, Shiina S, Teratani T, Obi S, Sato S, Koike Y (2005). Percutaneous radiofrequency ablation for hepatocellular carcinoma. An analysis of 1000 cases. Cancer.

[6] N’Kontchou G, Mahamoudi A, Aout M, Ganne-Carrie N, Grando V, Coderc E (2009). Radiofrequency ablation of hepatocellular carcinoma: long-term results and prognostic factors in 235 western patients with cirrhosis. Hepatology.

[7] Livraghi T, Meloni F, Di Stasi M, Rolle E, Solbiati L, Tinelli G (2008). Sustained complete response and complications rates after radiofrequency ablation of very early hepatocellular carcinoma in cirrhosis: is resection still the treatment of choice?. Hepatology.

[8] Tabuse K (1979). A new operative procedure of hepatic surgery using a microwave tissue coagulator. Arch Jpn Chir.

[9] Saitsu H, Yoshida M, Taniwaki S, Sato H, Okami N, Okuda Y (1991). Laparoscopic coagulo-necrotic therapy using Microtase for small hepatocellular carcinoma. Jpn J Gastroenterol.

[10] Seki T, Wakabayashi M, Nakagawa T, Imamura M, Tamai T, Nishimura A (1999). Percutaneous microwave coagulation therapy for patients with small hepatocellular carcinoma. Cancer.

[11] Rossi S, Di Stasi M, Buscarini E, Cavanna L, Quaretti P, Squassante E (1995). Percutaneous radio frequency interstitial thermal ablation in the treatment of small hepatocellular carcinoma. Cancer J Sci Am.

[12] Shibata T, Iimuro Y, Yamamoto Y, Maetani Y, Ametani F, Itoh K (2002). Small hepatocellular carcinoma: comparison of radio-frequency ablation and percutaneous microwave coagulation therapy. Radiology.

[13] Liang P, Wang Y (2007). Microwave ablation of hepatocellular carcinoma. Oncology.

[14] Liang P, Dong B, Yu X, Yu D, Wang Y, Feng L (2005). Prognostic factors for survival in patients with hepatocellular carcinoma after percutaneous microwave ablation. Radiology.

[15] Martin RCG, Scoggins CR, McMasters KM (2010). Safety and efficacy of microwave ablation of hepatic tumors: a prospective review of a 5-year experience. Ann Surg Oncol.

[16] Bhardwaj N, Strickland A, Ahmed F, El-Abassy M, Morgan B, Robertson GSM (2010). Microwave ablation for unresectable hepatic tumours: clinical results using a novel microwave probe and generator. Eur J Surg Oncol.

[17] Skinner MG, Iizuka MN, Kolios MC, Sherar MD (1998). A theoretical comparison of energy sources—microwave, ultrasound and laser—for interstitial thermal therapy. Phys Med Biol.

[18] Wright AS, Sampson LA, Warner TF, Mahvi AM, Lee FT (2005). Radiofrequency versus microwave ablation in a hepatic porcine model. Radiology.

[19] Takada Y, Kurata M, Ohkohchi N (2003). Rapid and aggressive recurrence accompanied by portal tumor thrombus after radiofrequency ablation for hepatocellular carcinoma. Int J Clin Oncol.

[20] Llovet JM, Vilana R, Bru C, Bianchi L, Salmeron JM, Boix L (2001). Increased risk of tumor seeding after percutaneous radiofrequency ablation for single hepatocellular carcinoma. Hepatology.

[21] Ruzzenente A, Manzoni GD, Molfetta M, Pachera S, Genco B, Donataccio M (2004). Rapid progression of hepatocellular carcinoma after radiofrequency ablation. World J Gastroenterol.

[22] Kotoh K, Morizono S, Kohjima M, Enjoji M, Sakai H, Nakamuta M (2005). Evaluation of liver parenchymal pressure and portal endothelium damage during radio frequency ablation in an in vivo porcine model. Liver Int.

[23] Kawamoto C, Yamauchi A, Baba Y, Kaneko K, Yakabi K (2010). Measurement of intrahepatic pressure during radiofrequency ablation in porcine liver. J Gastroenterol.

[24] Okusaka T, Okada S, Ueno H, Ikeda M, Shimada K, Yamamoto J (2002). Satellite lesions in patients with small hepatocellular carcinoma with reference to clinicopathologic features. Cancer.

[25] Wright AS, Lee FT, Mahvi DM (2003). Hepatic microwave ablation with multiple antennae results in synergistically larger zones of coagulation necrosis. Ann Surg Oncol.

[26] Simon CJ, Dupuy DE, Iannitti DA, Lu DSK, Yu NC, Asward BI (2007). Intraoperative triple antenna hepatic microwave ablation. AJR Am J Roentgenol.

[27] Lencioni RA, Allgaier HP, Cioni D, Olschewski M, Deibert P, Crocetti L (2003). Small hepatocellular carcinoma in cirrhosis: randomized comparison of radio-frequency thermal ablation versus percutaneous ethanol injection. Radiology.

[28] Ikeda M, Okada S, Ueno H, Okusaka T, Kuriyama H (2001). Radiofrequency ablation and percutaneous ethanol injection in patients with small hepatocellular carcinoma: a comparative study. Jpn J Clin Oncol.

[29] Kudo M, Chung H, Osaki Y (2003). Prognostic staging system for hepatocellular carcinoma (CLIP score): its value and limitations, and a proposal for a new staging system, the Japan Integrated Staging Score (JIS score). J Gastroenterol.

[30] Takayasu K, Arii S, Ikai I, Omata M, Okita K, Ichida T (2006). Prospective cohort study of transarterial chemoembolization for unresectable hepatocellular carcinoma in 8510 patients. Gastroenterology.

[31] Liang P, Wang Y, Yu X, Dong B (2009). Malignant liver tumors: treatment with percutaneous microwave ablation—complications among cohort of 1136 patients. Radiology.

